# Supplementary material for: The Rab Geranylgeranyl Transferase Beta Subunit Is Essential for Embryo and Seed Development in Arabidopsis thaliana
Source: Int J Mol Sci. 2021 Jul 24;22(15):7907. doi: 10.3390/ijms22157907 (PMC8347404; doi:10.3390/ijms22157907)
Supplement: Supplementary file 1 [file ijms-22-07907-s001.zip › ijms-1304096-supplementary.pdf]

Supplementary Figures:

Fig. S1 Development of stigmatic papillae and anthers in *rgtb1* plants.

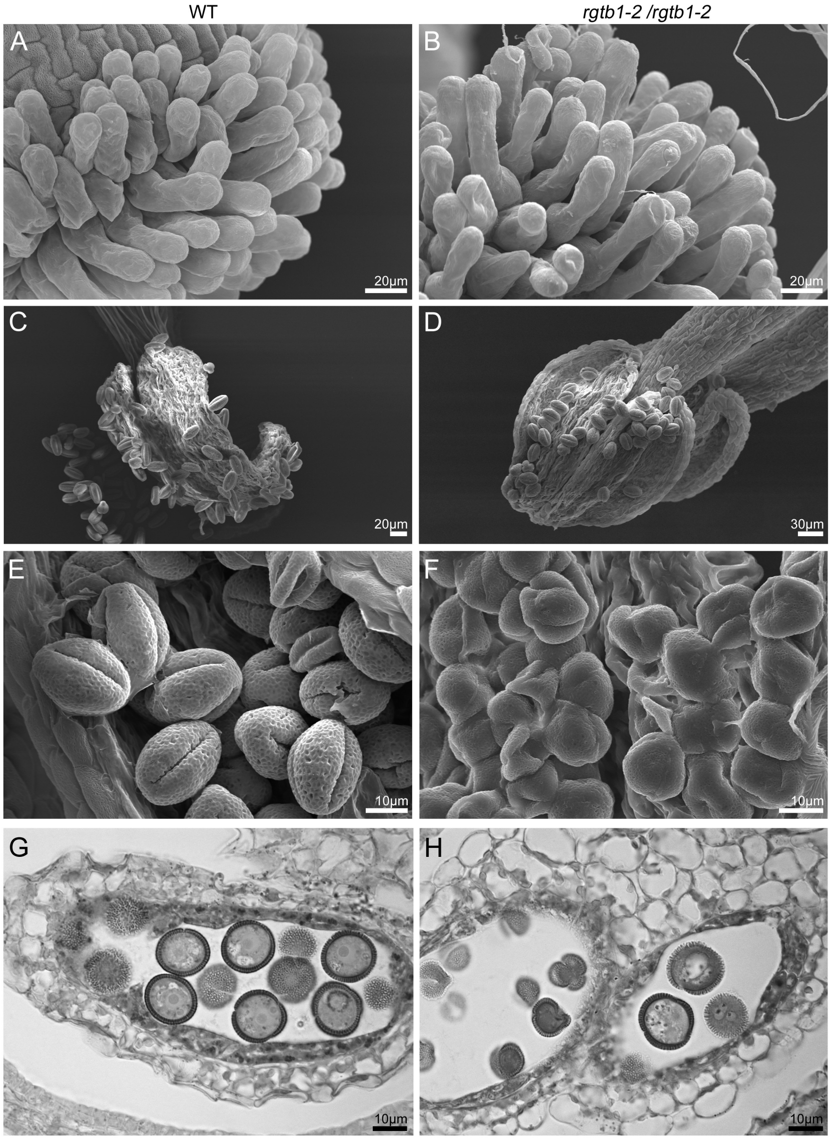

Stigmatic papillae developed correctly in *rgtb1* plants (**B**) in comparison to WT plants (**A**). SEM images of fixed flowers just before anthesis, scale bar corresponds to 20 μm for images **A,B**.

Anthers of WT (**C**) were bigger than in *rgtb1* (**D**) and opened fully upon dehiscence. Pollen grains were sticking more strongly to the interior of the locule, even in opened anthers in *rgtb1* (**F**) in comparison to WT (**E**). SEM images of fixed anthers at anthesis, scale bar corresponds to 20 μm (**C,E,F**) and 30 μm (**D**).

Tapetum development was delayed in *rgtb1* plants, while in WT one even layer of tapetum cells pad the anther locule (**G**) in *rgtb1* plants multilayered or uneven tapetum layer is observed (**H**). **G-H**, Semi-thin sections, light microscopy, scale bar corresponds to 10 μm .

Fig. S2 Quantification of incorrectly developing embryos and seeds in *rgtb1* plants.

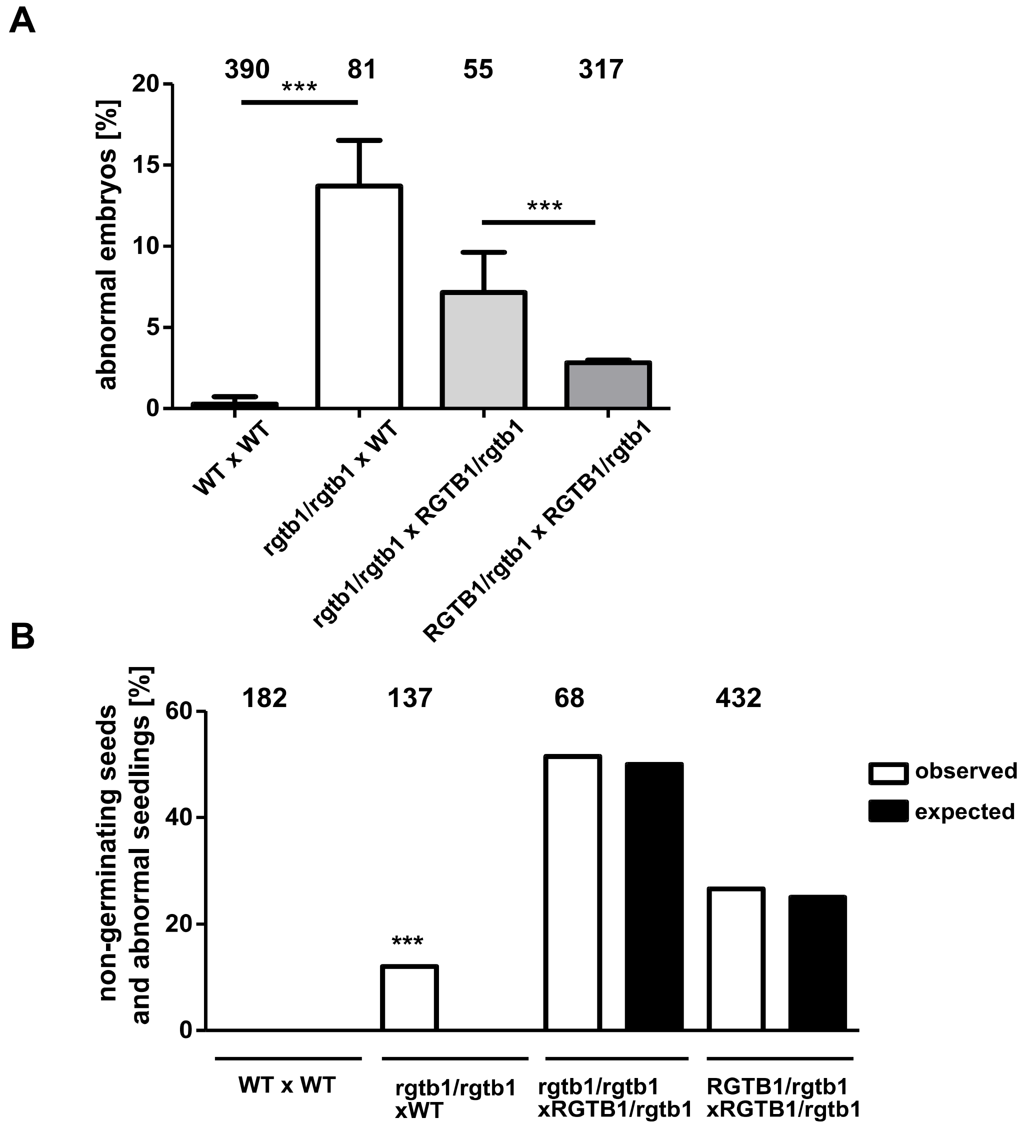

A, graph showing the fractions of abnormal embryo development on *rgtb1* plants. Embryo deformations counted included widening, shortening of the cotyledons, asymmetry, examples are shown on Fig. 6. Bars represent fraction of deformed embryos in siliques coming from plants from three independent cultivations, mean +/- SD. Values were compared with z-test for two proportins, *** denotes p value<0.001. Numbers above the bars indicate number of embryos counted for each cross. **B**, graph showing the fraction of non-functional seeds in *rgtb1* plants. Non-germinating seeds and deformed seedlings were counted together and compared to a theoretical fraction of *rgtb1/rgtb1* progeny (known to grow into dwarf deformed plants at maturity). Bars represent fraction of non-functional progeny in a given cross, *** denote p-value<0.001 in χ^2 comparison of obtained versus expected value, other comparisons gave insignificant results. Numbers above the bars indicate number of embryos counted for each cross.

Fig. S3 Transcriptomic analysis of RGT components in different seed compartments.

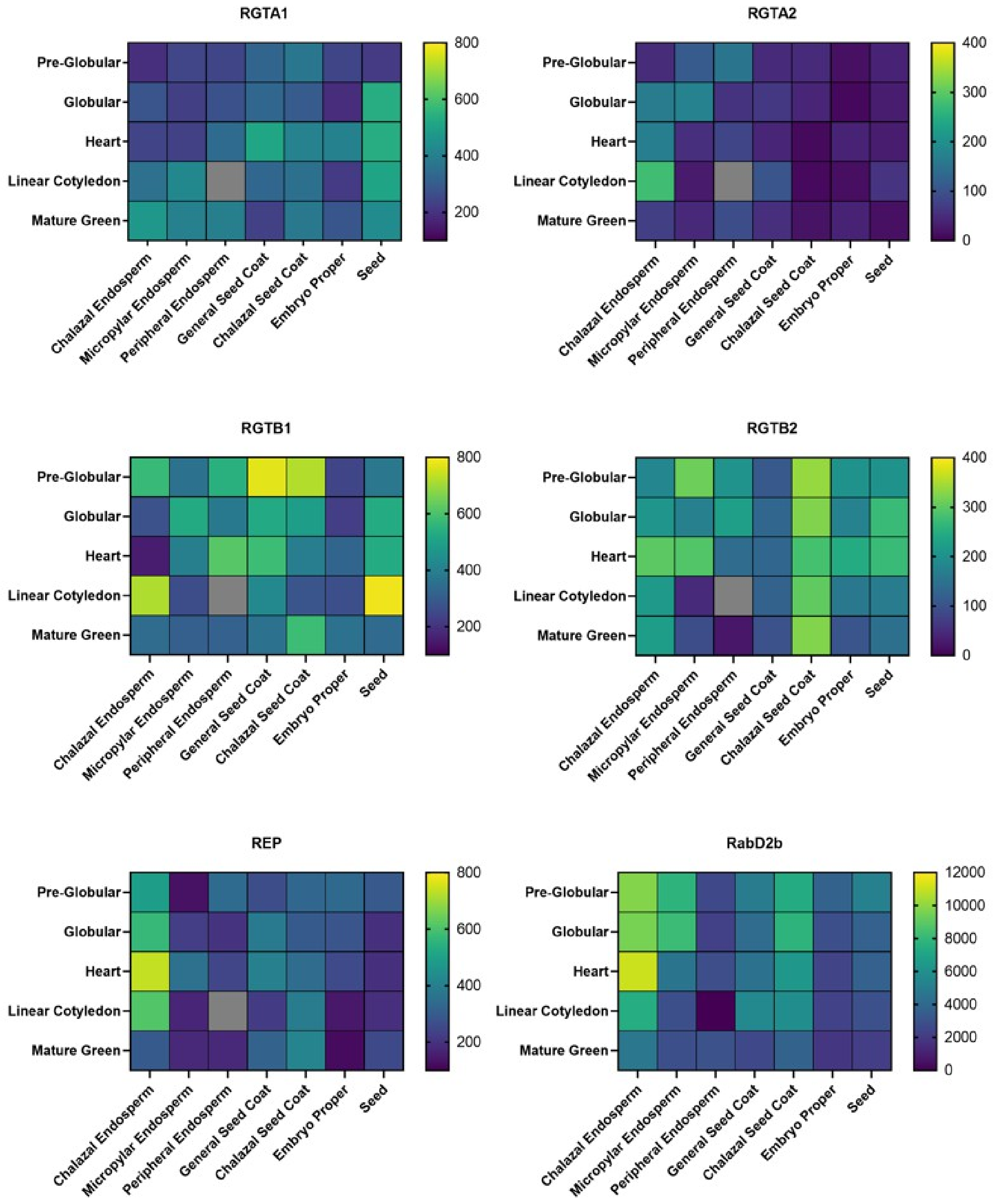

In silico expression profiles for RGT components encoding genes and ubiquitously expressed Rab D2b gene for comparison were extracted from [102]. Data was obtained from http://seedgenenetwork.net/, which incorporates microarray profiles of laser dissected *Arabidopsis* seed compartments at different stages of development. The stages of development include: Pre-globular, Globular, Heart, Linear Cotyledon, Green cotyledon The tissues include: Chalazal Endosperm, Chalazal Seed Coat, Embryo Proper, General Seed Coat, Micropylar Endosperm, Peripheral Endosperm, Whole Seed.
